# Atrial High-Rate Episodes Detected by Cardiac Implantable Electronic Devices: Dynamic Changes in Episodes and Predictors of Incident Atrial Fibrillation

**DOI:** 10.3390/biology11030443

**Published:** 2022-03-15

**Authors:** Jacopo Francesco Imberti, Niccolò Bonini, Alberto Tosetti, Davide Antonio Mei, Luigi Gerra, Vincenzo Livio Malavasi, Andrea Mazza, Gregory Y. H. Lip, Giuseppe Boriani

**Affiliations:** 1 Department of Biomedical, Metabolic and Neural Sciences, Cardiology Division, University of Modena and Reggio Emilia, Policlinico di Modena, 41125 Modena, Italy; jacopo.imberti@hotmail.it (J.F.I.); bonini.niccolo93@gmail.com (N.B.); albertotosetti1@gmail.com (A.T.); davide.mei93@gmail.com (D.A.M.); luigi.gerra@gmail.com (L.G.); nanni.malavasi@gmail.com (V.L.M.); 2 Clinical and Experimental Medicine PhD Program, University of Modena and Reggio Emilia, 41125 Modena, Italy; 3 Liverpool Centre for Cardiovascular Science, University of Liverpool and Liverpool Heart & Chest Hospital, Liverpool L10 0AD, UK; gregory.lip@liverpool.ac.uk; 4 Cardiology Division, S. Maria della Stella Hospital, 05018 Orvieto, Italy; and.mazza_m@libero.it

**Keywords:** atrial high-rate episodes, AHRE, atrial fibrillation, subclinical atrial fibrillation, cardiac implantable electronic devices, pacemaker, defibrillators

## Abstract

**Simple Summary:**

Atrial high rate episodes (AHRE) detected by cardiac implantable electronic devices (CIEDs) may be associated with a risk of progression towards long-lasting episodes (≥24 h) and clinical atrial fibrillation (AF). Identification of this subset of patients is key to promptly starting oral anticoagulant therapy and possibly avoiding stroke and systemic thromboembolism. In the present study, we describe the clinical characteristics of 104 retrospectively enrolled patients with AHRE episodes lasting 5 min–23 h 59 min, no AF at 12-lead ECG, and no history of prior clinical AF. Moreover, we define predictors of the composite outcome of clinical AF and/or AHRE episodes lasting ≥24 h. Over a median follow-up of 24.3 (10.6–40.3) months, 31/104 (29.8%) patients experienced the composite outcome. Baseline CHA_2_DS_2_-VASc score and the longest AHRE episode at enrollment lasting 12 h–23 h 59 min were independently associated with the composite outcome and with incident clinical AF as well. To conclude, in patients with AHRE, the incidence of clinical AF or AHRE episodes lasting ≥24 h is high. Baseline patients’ characteristics (CHA_2_DS_2_-VASc score) and AHRE duration may help to intensify monitoring and decision-making, being independently associated with clinical AF in a two-year follow-up.

**Abstract:**

Background. Atrial high rate episodes (AHRE) detected by cardiac implantable electronic devices (CIEDs) may be associated with a risk of progression towards long-lasting episodes (≥24 h) and clinical atrial fibrillation (AF). Methods. Consecutive CIED patients presenting AHRE (with confirmation of an arrhythmia lasting 5 min–23 h 59 min, atrial rate ≥175/min, with no AF at 12-lead ECG and no prior clinical AF) were retrospectively enrolled. The aims of this study were to describe patients’ characteristics and the incidence of adverse events, and second, to identify potential predictors of the composite outcome of clinical AF and/or AHRE episodes lasting ≥24 h. Results. 104/107 (97.2%) patients (median age 79.7 (74.0–84.2), 33.7% female) had available follow-up data. Over a median follow-up of 24.3 (10.6–40.3) months, 31/104 (29.8%) patients experienced the composite outcome of clinical AF or AHRE episodes lasting ≥24 h. Baseline CHA_2_DS_2_-VASc score and the longest AHRE episode at enrollment lasting 12 h–23 h 59 min were independently associated with the composite outcome (Hazard ratio (HR); 95% CI: 1.40; 1.07–1.83 and HR: 8.15; 95% CI 2.32–28.65, respectively). Baseline CHA_2_DS_2_-VASc score and the longest AHRE episode at enrollment lasting 12 h–23 h 59 min were the only independent predictors of incident clinical AF (HR: 1.45; 95% CI 1.06–2.00 and HR: 4.25; 95% CI 1.05–17.20, respectively). Conclusions. In patients with AHRE, the incidence of clinical AF or AHRE episodes lasting ≥24 h is high in a two-year follow-up. Baseline patients’ characteristics (CHA_2_DS_2_-VASc score) and AHRE duration may help to intensify monitoring and decision-making, being independently associated with clinical AF at follow-up.

## 1. Introduction

Atrial high rate episodes (AHRE) are a relatively new clinical entity currently defined as episodes of atrial tachycardia lasting ≥5 min with an atrial rate ≥175/min detected through the capability of continuous monitoring and data storage of cardiac implantable electronic devices (CIEDs) with atrial sensing, occurring in patients with no history of clinical atrial fibrillation (AF) or AF related symptoms, and with no AF at routine 12-lead ECG [1,2]. Recent studies derived from trials or large observational cohorts show that daily AHRE burden tends to increase over time [3,4], exceeding the 23–24 h threshold in up to one-fourth of patients, and are associated with a higher risk of systemic thromboembolism as compared to shorter episodes [5,6]. Similarly, AHRE are associated with a three to five-fold higher risk of incident clinical AF [7,8], a cardiac arrhythmia significantly related with an excess in morbidity, mortality, and increased healthcare costs [9,10]. In this context, robust predictors of transition to long-lasting AHRE/clinical AF are lacking but they are clinically needed in order to possibly identify those at risk of disease progression through a more intense follow-up and remote monitoring, as well as facilitate decision-making on institution of anticoagulant prophylaxis [11], as part of a holistic or integrated approach to AF care [12,13].

In the current study, we report on the clinical characteristics and clinical course of patients with AHRE lasting more than 5 min but less than 24 h in order to identify potential risk factors for transition to long-lasting AHRE and/or progression to clinical AF. 

## 2. Methods

We performed a retrospective observational study enrolling consecutive patients with a CIED-detected AHRE presenting at our institution, a tertiary care academic hospital, between 1 January 2016 and 30 September 2021. Inclusion criteria were as follows: (1) Permanent PM, ICD, or CRT-P/D (but not loop recorder) capable of detecting AHRE, (2) at baseline assessment, documentation of at least one episode of AHRE of 5 min-23 h 59 min in duration with average atrial rate ≥ 175/min documented by the CIED at any time prior to enrollment visit, (3) age ≥18 years. CIED-recorded AHRE tracings were visually inspected and adjudicated by two cardiologists (JFI and GB) independently to avoid false positives. Disagreement was resolved by consensus-based discussion. The only exclusion criterion was clinical AF documented by surface ECG (12 lead ECG) or an ECG strip (Telemetry, Holter) lasting ≥30 s. Data were obtained from electronic and paper medical records, including PM/ICD follow-up charts and documented printout of device interrogations. Total interatrial block (IAB) was defined as a biphasic P wave morphology in leads DII-DII-aVF and duration ≥120 ms [14]. According to its original definition [15], the CHA_2_DS_2_-VASc score was defined as follows: Congestive heart failure (1 point), hypertension (1 point), age ≥75 years (2 points) or age ≥65 years (1 point), diabetes (1 point), stroke (2 points), vascular disease (1 point), and female sex (1 point) for a total of maximum nine points. The study protocol (No. AOU 0015015/21) was approved by the local IRB/Ethics Committee in compliance with national regulations.

The aims of the present study were as follows: (i) To describe patients’ characteristics and the incidence of adverse events of a contemporary cohort of patients with AHRE, (ii) to identify potential predictors of the composite outcome of clinical AF detected by surface ECG and longest AHRE episode ≥24 h, and (iii) to investigate potential predictors of the disaggregate outcomes.

### Statistical Analysis

Continuous variables were expressed as mean ± standard deviation or median with interquartile range (IQR) for skewed distributions. Categorical variables were expressed as counts and percentages. Survival curves were built using the Kaplan–Meier method. Incidence rates were calculated by dividing the number of patients reaching the outcome by the total number of person-months. Variables potentially associated with the outcomes were tested using Cox’s univariate regression analysis. Characteristics significantly (*p* < 0.05) associated with the outcomes were thereafter used to build a multivariate Cox’s proportional hazards regression model. A *p* value < 0.05 was considered statistically significant in all the analyses. Analyses were performed using SPSS^®^ version 26 (IBM Corp, Armonk, NY, USA).

## 3. Results

### 3.1. Study and Population Characteristics

Overall, 104/107 (97.2%) patients had available follow-up data and were included in the analysis. Median age was 79.7 (74.0–84.2) months and 35/104 (33.7%) were female. Median CHA_2_DS_2_VASc score was 4 (3–5) and 3.2% were treated with class I or III antiarrhythmic drugs for control of atrial or ventricular ectopics. Baseline patients’ characteristics are summarized in Table 1.

PM was the most common type of CIED implanted (78.8%), followed by CRT-P/D (15.4%) and ICD (5.8%). The most common pacing mode at implant was DDD (73.1%) and the most frequent reason for implant was atrioventricular block (51.9%) (Supplemental Table S1). Median age at implant was 75.6 (70.6–80.5) years and median time from CIED implant to enrollment was 2.2 (1.2–5.0) years (Supplemental Table S1). Overall, 39.2% of patients had the longest AHRE episode at enrollment lasting <1 h, 53.9% between 1 h and 11 h 59 min, and 6.9% between 12 h and 23 h 59 min.

### 3.2. Study Outcomes

**Figure 1:**
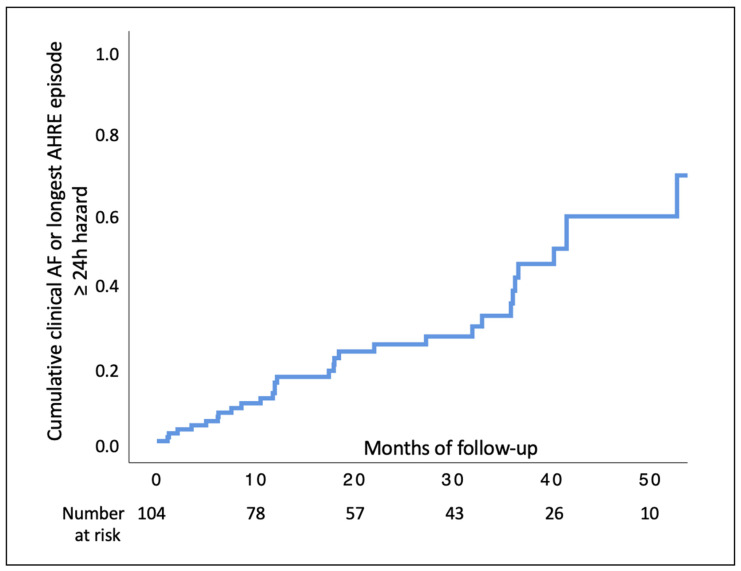
Kaplan–Meier curve for the composite outcome. Shown are estimates of the probability of the primary composite end point (clinical atrial fibrillation or longest AHRE episode ≥24 h in duration). AF, atrial fibrillation.

Over a median period of 24.3 (10.6–40.3) months, 31/104 (29.8%) patients experienced the composite outcome of clinical AF or single longest AHRE episode ≥24 h, with an incidence of 11.54 per 100 person-months (31 first events over 2687.21 person-month). The cumulative incidence of this composite outcome at the Kaplan–Meier curve is shown in Figure 1. At multivariate analysis, the CHA_2_DS_2_-VASc score at baseline and a longest AHRE episode at enrollment lasting 12 h–23 h 59 min were independently associated with the composite outcome (HR; 95% confidence interval (CI) 1.40; 1.07–1.83 and HR 8.15; 95% CI 2.32–28.65 respectively) (Table 2). 

**Figure 2:**
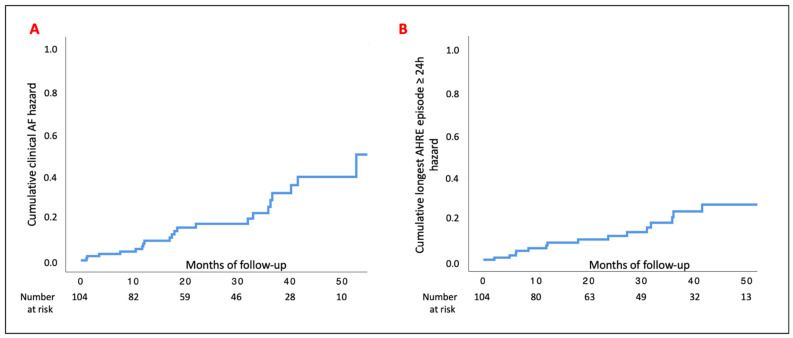
Kaplan–Meier curves. Shown are estimates of the probability of clinical atrial fibrillation (Panel **A**) and single longest AHRE episode ≥24 h in duration (Panel **B**). AF, atrial fibrillation.

Incident clinical AF occurred during follow-up in 21/104 (20.2%) patients over a median period of 26.1 (12.0–41.2) months. The incidence was 7.50 per 1000 person-months (21 first events over 2801.52 person-month). The Kaplan–Meier curve is shown in Figure 2, Panel A. A longest AHRE episode ≥24 h occurred in 16/104 (15.4%) patients over a median period of 29.6 (12.0–42.4) months. The incidence was 5.92 per 1000 person-months (16 first events over 2944.63 person months). The Kaplan–Meier curve is shown in Figure 2, Panel B. At multivariate analysis, the CHA_2_DS_2_-VASc score at baseline and the longest AHRE episode at enrollment lasting 12 h–23 h 59 min were independently associated with clinical AF (HR 1.45; 95% CI 1.06–2.00 and HR 4.25; 95% CI 1.05–17.20, respectively) (Supplemental Table S2). On the other hand, no predictors of single longest AHRE episode ≥24 h were detected (Supplemental Table S3).

## 4. Discussion

In the present paper, we provide clinical insights on a population of patients with AHRE, reflecting the definition provided by the latest ESC guidelines [1]. Our principal finding was that CHA_2_DS_2_-VASc score at baseline and the longest AHRE episode at enrollment lasting 12 h–23 h 59 min were independently associated with both the composite outcome (clinical AF and single longest AHRE episode ≥24 h) and incident clinical AF.

Few studies have addressed the issue of the natural history of AHRE [3,4,16]. Boriani et al. [3], in an analysis based on a dataset including patients from multicenter registries and a trial, found that 24% of patients with AHRE transitioned from a lower threshold to a daily AHRE burden of ≥23 h during a mean follow-up of 2.4 ± 1.7 years. Patients with higher daily AHRE burden at baseline and CHADS_2_ score ≥2 transitioned faster to longer episodes. In another study [4], 205 patients with AHRE detected by implantable loop recorder were followed for a median of 40.2 months: 16% of patients developed AHRE episodes lasting ≥24 h and no variable was found associated with these episodes. Interestingly, most patients were asymptomatic both at AHRE onset (90%) and during further monitoring (87%), a finding suggesting that better risk predictors are needed to diagnose and treat AF before it manifests as stroke. 

Our study provides simple clinical parameters that allow the identification of patients at higher risk of incident clinical AF and/or long-lasting AHRE episodes which may warrant closer follow-up visits. In consideration of the risk of stroke/thromboembolic events associated with asymptomatic clinical AF, not different from the risk associated with symptomatic clinical AF [17], prompt initiation of anticoagulation is required in patients at risk who develop clinical AF during follow-up, independently on symptoms, and this stresses the value of close monitoring of patients presenting with AHRE, also with the aid, if possible, of remote monitoring [18]. We found that CHA_2_DS_2_-VASc score was independently associated with the composite outcome of clinical AF and longest AHRE episode ≥24 h and also predicted clinical AF per se. The CHA_2_DS_2_-VASc score is a well acknowledged risk score for thromboembolism in AF but is also associated with incident AF and AF recurrence prediction [19,20]. Our study reinforces the usefulness of this simple clinical score, based only on readily available clinical parameters, and adds evidence that longer episodes of AHRE are associated with a greater risk of evolution toward long-lasting episodes (≥24 h) and clinical AF. No predictor of AHRE episodes lasting ≥24 h was found, possibly due to the relatively small sample size and low event rate, which limited the statistical power of the study. In this context, remote monitoring and dedicated smartphone applications may be a valuable tool to better characterize the dynamic changes of AHRE episodes, support timely decision-making on patients’ therapy and reduce in-person follow-up visits [18,21].

Our patient population is mainly characterized by patients implanted with a pacemaker, and with this regard is related to an older patients’ population as compared to other reports [3,22,23]. The difference in the type of implanted device is associated with differences in patients’ age [24] and probably also in the availability of remote monitoring for patients’ follow-up. Indeed, even if remote monitoring is currently recommended as a standard for follow-up not only for defibrillators and devices for cardiac resynchronization therapy but also for pacemakers [18,25], it has not been constantly applied for the latter devices [26,27]. Even if the rate of implementation of remote monitoring has markedly increased after the COVID-19 pandemic [28,29,30], our data suggest that pacemaker patients with higher CHA_2_DS_2_-VASc should be prioritized for being remotely monitored in order to promptly detect the progression of AHRE. This may be important for taking into consideration in selected cases, on an individual basis, improved patient characterization and the prescription of oral anticoagulants, as suggested by the recent ESC Guidelines on AF [1,13]. Indeed, there is still uncertainty on how to manage the thromboembolic risk associated with AHRE [1,8,31] before the development of clinical AF, and two randomized trials on this topic are currently ongoing [32,33]. In view of the lack of a well-defined strategy for managing the risk of stroke, the prevention of stroke was not a specific focus of evaluation in the present study. The individual predisposition to progress towards clinical AF and the increased risk of thromboembolism, which is almost independent of the presence of the arrhythmia itself, may suggest that AHRE can be interpreted as a marker of underlying atrial cardiomyopathy [34,35]. This clinical entity has been recently defined as “any complex of structural, architectural, contractile, or electrophysiological changes affecting the atria with the potential to produce clinically-relevant manifestations”. The histopathologic findings observed in the atrial cardiomyopathy encompass cardiomyocyte changes, fibrotic changes, combined cardiomyocyte-pathology/fibrosis, and non-collagen infiltration, which may be both the trigger and/ or the consequence of AF [36]. In this context, structural and electrophysiologic changes may start a vicious circle that promotes, maintains, and progresses AF [37,38,39]. In our study, we highlight that AHRE progression towards clinical AF or long-lasting episodes is related to patient specific clinical risk factors, streamlined in the CHA_2_DS_2_-VASc score, but also to the duration of baseline AHRE episodes. Thus, longer episodes may contribute to or be a red flag for deranged atria. Other indices of atrial cardiomyopathy, such as echocardiographic or MRI data, will be valuable to further explain the complex pathophysiology underlying atrial cardiomyopathy and its multiple interactions with AHRE and clinical AF [40,41,42]. Recent AF guidelines [1] emphasize the role of an integrated and multidisciplinary approach to AF patients care, streamlined in the ABC (Atrial Fibrillation Better Care) pathway. The ‘A’ domain refers to Avoid stroke with anticoagulation, ‘B’ to Better symptom management with patient-centered and symptom-directed decisions on rate or rhythm control, and ‘C’ to comorbidity and cardiovascular risk factor management, including lifestyle changes [13]. One of the key features of this pathway is to increase physicians’ awareness on the management of comorbidities in a simple way, and its benefits have been extensively proved in different patient populations [43,44,45]. In the mAFA II prospective cluster-randomized trial, 1646 patients were allocated to a mobile health technology-supported AF integrated management strategy (including the ABC pathway), and 1678 patients were allocated to usual care. The authors found that rates of the composite outcome of ischemic stroke/systemic thromboembolism, death, and rehospitalization were lower in the intervention arm (1.9% vs. 6.0%; HR: 0.39; 95% CI 0.22–0.67) [46]. These results were subsequently confirmed in the long-term extension cohort as well [47]. In a systematic review of eight studies, the pooled prevalence of ABC-adherent management was low (21%). Patients treated according to the ABC pathway had a significant reduction in the risk of all-cause death (58%), cardiovascular death (63%), stroke (45%), and major bleeding (31%) as compared with ABC non-adherent patients [12]. Finally, Guo et al. [48] provided further insights on the clinical impact of a structured AF characterization and management. Six thousand four hundred nineteen AF patients were retrospectively subdivided into three groups. Group 1 included patients not characterized using the 4S-AF scheme and not managed according to the ABC pathway (59.8%), group 2 included patients characterized according to the 4S-AF scheme but ABC pathway non-adherent or vice versa, and group 3 included patients characterized according to the 4S-AF scheme and ABC pathway adherent (17.4%). Patients in groups 2 and 3 showed lower odds of the composite endpoint of all-cause death/ any thromboembolic event (OR: 0.28; 95% CI 0.20–0.37 and OR: 0.19; 95% CI 0.12–0.31 respectively) as compared to group 1. This evidence suggests that addressing comorbidities favorably impact patients’ outcomes. Similarly, it is reasonable to hypothesize that a holistic approach to AHRE patients’ management may ameliorate their prognosis and slow the disease’s progression down. Further studies are needed to shed light on this important aspect of AHRE management. 

## 5. Limitations

Our cohort represents a single-medical center experience with small sample size, thus limiting the generalizability of the results. As with any retrospective study, the statistical power of the analysis is limited due to the absence of a prospective design and follow-up and did not allow us to investigate possible predictors of single longest AHRE episode ≥24 h. Although the multivariate analysis was adjusted for many well-known risk factors for incident clinical AF some are missing, and residual confounders cannot be completely ruled out. Hard outcomes, such as death, systemic thromboembolism, acute coronary syndromes, and cardiovascular hospital admissions, were not evaluated. 

## 6. Conclusions

In a contemporary real-world cohort of patients with AHRE episodes lasting ≥5 min but less than 24 h, the incidence of first diagnosed clinical AF or single longest AHRE episode lasting ≥24 h is high. Patient characteristics at baseline (CHA_2_DS_2_-VASc score) and AHRE duration are independently associated with clinical AF at follow-up and may help to intensify patient monitoring for timely decision-making and management.

## Figures and Tables

**Table 1:** Baseline patients’ characteristics. AADs, antiarrhythmic drugs; ACEi, angiotensin-converting enzyme inhibitors; ARB, angiotensin receptor blockers; ARNI, Angiotensin Receptor Neprilysin Inhibitor; CKD, chronic kidney disease; IAB, interatrial block; MRA, mineralocorticoid receptor antagonist. * Median (interquartile range).

	N (%) (*n* = 104)
Age *	79.7 (74.0–84.2)
Female sex	35 (33.7)
Heart failure	27 (26.0)
Diabetes	36 (34.6)
Stroke	8 (7.7)
CAD	31 (29.8)
PAD	16 (15.4)
Hypertension	87 (83.7)
CKD (*n* = 86)	40 (46.5)
CHA_2_DS_2_-VASc *	4 (3–5)
Total IAB (*n* = 102)	7 (6.9)
Class I/III AADs (*n* = 95)	3 (3.2)
Beta-blockers (*n* = 96)	55 (57.3)
ACEi/ARB/ARNI (*n* = 96)	55 (57.3)
MRA (*n* = 96)	13 (12.5)
Statin (*n* = 96)	52 (54.2)

**Table 2:** Univariate and multivariate Cox’s regression analysis for the composite outcome of clinical atrial fibrillation or single longest AHRE episode ≥24 h in duration. CI, confidence interval; CKD, chronic kidney disease; HR, hazard ratio; IAB, interatrial block.

	Univariate			Multivariate		
	HR	*p*-Value	CI	HR	*p*-Value	CI
Age	1.00	0.88	0.96–1.05			
Age ≥ 80 years	1.23	0.56	0.61–2.50			
Female sex	0.74	0.45	0.35–1.61			
CHA_2_DS_2_-VASc	1.41	<0.01	1.10–1.81	1.40	0.01	1.07–1.83
CKD	1.24	0.57	0.59–2.57			
Pacing mode at implant						
DDD	Ref.					
VVI	1.10	0.92	0.14–8.56			
AAI	1.48	0.53	0.43–5.06			
VDD	0.86	0.76	0.32–2.30			
Total IAB	1.91	0.24	0.66–5.53			
Longest AHRE episode at enrollment						
5–59 min	Ref.			Ref.		
1 h–11 h 59 min	1.74	0.18	0.78–3.88	1.52	0.31	0.68–3.43
12 h–23 h 59 min	7.94	<0.01	2.27–27.79	8.15	<0.01	2.32–28.65

## Data Availability

Data will be shared upon reasonable request and IRB approval.

## Supplementary Materials

The following supporting information can be downloaded at: https://www.mdpi.com/article/10.3390/biology11030443/s1, Table S1: Implanted CIEDs characteristics. Table S2: Univariate and multivariate Cox’s regression analysis for clinical atrial fibrillation. Table S3: Univariate Cox’s regression analysis for single AHRE episode ≥24 h in duration.
