# Supplementary material for: Atrial High-Rate Episodes Detected by Cardiac Implantable Electronic Devices: Dynamic Changes in Episodes and Predictors of Incident Atrial Fibrillation
Source: Biology (Basel). 2022 Mar 15;11(3):443. doi: 10.3390/biology11030443 (PMC8945035; doi:10.3390/biology11030443)
Supplement: Supplementary file 1 [file biology-11-00443-s001.zip › biology-1557279-supplementary.pdf]

Atrial High-Rate Episodes detected by Cardiac Implantable Electronic Devices: Dynamic changes in episodes and predictors of incident atrial fibrillation

Jacopo Francesco Imberti[1,2,3], Niccolò Bonini[1], Alberto Tosetti[1], Davide A. Mei[1], Luigi Gerra[1], Vincenzo Livio Malavasi[1], Andrea Mazza[4], Gregory Y. H. Lip[3], Giuseppe Boriani[1*]

[1]Cardiology Division, Department of Biomedical, Metabolic and Neural Sciences, University of Modena and Reggio Emilia, Policlinico di Modena, Modena, Italy.
[2]Clinical and Experimental Medicine PhD Program, University of Modena and Reggio Emilia, Modena, Italy
[3]Liverpool Centre for Cardiovascular Science, University of Liverpool and Liverpool Heart & Chest Hospital, Liverpool, United Kingdom
[4] Cardiology Division, S. Maria della Stella Hospital, Orvieto, Italy.

Supplemental Table S1. Implanted CIEDs characteristics.

Supplemental Table S2. Univariate and multivariate Cox's regression analysis for clinical atrial fibrillation.

Supplemental Table S3. Univariate Cox's regression analysis for single AHRE episode ≥ 24h in duration.

	**N (%)**<br>(n=104)
Age at implant (years)*	75.6 (70.6-80.5)
CIED implant to enrollment (years)*	2.2 (1.2-5.0)
CIED type	
PM	82 (78.8)
ICD	6 (5.8)
CRT-P/D	16 (15.4)
Implant reason	
Sick sinus syndrome	29 (27.9)
Atrioventricular block	54 (51.9)
Primary prevention and/or cardiac resynchronization therapy	21 (20.2)
Manufacturer	
Medtronic	59 (56.7)
St. Jude Medical	3 (2.9)
Boston Scientific	28 (26.9)
Sorin	9 (8.7)
Biotronik	5 (4.8)
Pacing mode at implant	
DDD	76 (73.1)
VVI	2 (1.9)
AAI	11 (10.6)
VDD	15 (14.4)

Supplemental Table S1. Implanted CIED characteristics. CRT-P/D, cardiac resynchronization therapy and pacing/defibrillator; ICD, implantable cardioverter defibrillator; PM, pacemaker.
*Median (interquartile range)

	Univariate			Multivariate		
	HR	p-value	CI	HR	p-value	CI
Age	1.01	0.71	0.96-1.06			
Age ≥ 80 years	1.30	0.54	0.55-3.07			
Female sex	0.85	0.72	0.34-2.10			
CHA_2DS_2-VASc	1.38	0.04	1.02-1.87	1.45	0.02	1.06-2.00
CKD	1.24	0.64	0.50-3.08			
Pacing mode at implant						
DDD		Ref.				
VVI	1.96	0.52	0.25-15.11			
AAI	0.70	0.73	0.09-5.37			
VDD	1.01	0.99	0.33-3.06			
Total IAB	1.53	0.57	0.35-6.67			
Longest AHRE episode at enrollment						
5-59min		Ref.			Ref.	
1h-11h59min	1.74	0.27	0.65-4.65	1.34	0.56	0.50-3.60
12h-23h59min	10.65	<0.01	2.42-46.80	4.25	0.04	1.05-17.20

Supplemental Table S2. Univariate and multivariate Cox's regression analysis for clinical atrial fibrillation.

CI, Confidence interval; CKD, chronic kidney disease; HR, hazard ratio; IAB, interatrial block.

	Univariate		
	HR	p-value	CI
Age	0.98	0.48	0.93-1.03
Age ≥ 80 years	0.62	0.36	0.23-1.71
Female sex	0.37	0.12	0.10-1.31
CHA_2DS_2-VASc	1.19	0.26	0.88-1.59
CKD	0.81	0.69	0.29-2.27
Pacing mode at implant			
DDD		Ref.	
VVI	0.00	0.99	0.00-
AAI	1.41	0.57	0.31-6.35
VDD	0.62	0.65	0.14-2.78
Total IAB	1.56	0.56	0.35-6.94
Longest AHRE episode at enrollment			
5-59min		Ref.	
1h-11h59min	1.53	0.45	0.51-4.60
12h-23h59min	3.74	0.12	0.71-19.64

Supplemental Table S3. Univariate Cox regression analysis for single AHRE episode ≥ 24h in duration.

CI, confidence interval; CKD, chronic kidney disease; HR, hazard ratio; IAB, interatrial block.
